# Cinnamon Bark Essential Oil as a Natural Plant Protection Agent: Chemical Profile, Antimicrobial Activity, and Defence Induction

**DOI:** 10.3390/molecules31061036

**Published:** 2026-03-20

**Authors:** Elżbieta Gębarowska, Karolina Budek, Martyna Gębarowska, Anna Kmieć, Antoni Szumny

**Affiliations:** 1 Wrocław University of Environmental and Life Sciences, Department of Plant Protection, Laboratory of Biogeochemistry and Environmental Microbiology, Grunwaldzka Str 53, 50-375 Wrocław, Poland; karolina.budek@upwr.edu.pl (K.B.); anna.kmiec@upwr.edu.pl (A.K.); 2 Wrocław University of Environmental and Life Sciences, Department of Biostructure and Animal Physiology, Veterinary Biotechnology Student Research Club “Refectio”, Kożuchowska Str 1, 51-631 Wrocław, Poland; 126695@student.upwr.edu.pl; 3 Wrocław University of Environmental and Life Sciences, Department of Food Chemistry and Biocatalysis, Norwida Str 25, 50-375 Wrocław, Poland; antoni.szumny@upwr.edu.pl

**Keywords:** *Cinnamomum verum*, cinnamon bark essential oil, chemical composition, phytopathogens, antimicrobial activity, MTT assay, PAL activity, plant defence elicitor, phytotoxicity, biopesticide

## Abstract

Ceylon cinnamon (*Cinnamomum verum* J. Presl) bark essential oil (CBO) represents a promising source of natural bioactive compounds for biological plant protection. For the first time, the antibacterial and antifungal activity of CBO was systematically evaluated against a curated panel of phytopathogenic strains (IOR collection), revealing broad-spectrum efficacy across both bacteria and filamentous pathogens. This study evaluated its chemical composition, antimicrobial activity against phytopathogens, effects on bacterial metabolic activity, and its ability to induce plant defence responses. CBO was dominated by cinnamaldehyde, linalool, and eucalyptol. The oil exhibited strong antibacterial activity against *Dickeya dadantii*, *Pectobacterium carotovorum*, *Pseudomonas syringae*, and *Xanthomonas hortorum* as well as antifungal activity against *Fusarium graminearum*, *F. culmorum*, *Rhizoctonia solani* and *Phytophthora cinnamomi*. Metabolic assays revealed a marked reduction in bacterial metabolic activity, indicating that CBO disrupts physiological processes and inhibits growth. In planta experiments showed that foliar application of CBO stimulated PAL activity in wheat leaves without visible phytotoxic symptoms. These findings demonstrate a multifunctional mode of action of CBO, combining direct antimicrobial effects with the elicitation of plant defence responses, and support its potential application in sustainable crop protection.

## 1. Introduction

Plant diseases represent one of the most serious threats to global agricultural production, significantly reducing yields and crop quality and posing risks to food security. Annual losses caused by plant pathogens are estimated to exceed 30–40% of global crop production, corresponding to substantial economic losses [1,2]. Moreover, the increasing resistance of phytopathogens to conventional plant protection agents, particularly to synthetic fungicides with narrow modes of action, further limits the effectiveness of chemical control and represents a major challenge for modern crop protection [3,4,5,6]. Consequently, there is an urgent need to develop more sustainable and environmentally friendly strategies, including natural bioactive compounds and integrated disease management approaches [7,8,9].

Natural chemical compounds offer diverse mechanisms of action that may complement or replace conventional pesticides while minimising environmental effects. Plant secondary metabolites, particularly essential oils, represent a rich source of bioactive substances with broad-spectrum antimicrobial activity [10,11]. Essential oils consist of volatile compounds such as aldehydes, phenols, monoterpenes, and sesquiterpenes, which can interact with microbial cell membranes, enzymatic systems, and metabolic pathways [12,13]. Numerous in vitro studies have demonstrated that plant-derived essential oils inhibit the growth of fungi and bacteria, supporting their potential use as natural fungicidal and bacteriostatic agents [8,14]. For example, cinnamon bark oil and its major constituent, cinnamaldehyde, have shown strong inhibitory effects in various in vitro systems. Although numerous studies have focused on clinically relevant human pathogens [15,16,17], considerably fewer investigations have addressed their effects on phytopathogenic bacteria and fungi relevant to crop protection [11,13]. Recent reports indicate that essential oils may inhibit plant pathogens through membrane disruption, interference with enzymatic systems, and modulation of oxidative balance, suggesting their potential as natural plant protection agents in sustainable agriculture [8,14]. However, relatively few studies have investigated the direct application of cinnamon bark essential oil (CBO) in plant protection or its effects on phytopathogenic bacteria and fungi, highlighting a research gap between medical microbiology and plant pathology [10,11]. Moreover, most available studies rely mainly on classical growth-based assays and focus on a limited range of microorganisms, while data concerning metabolic-level effects and the induction of plant defence mechanisms remain scarce [18,19].

In addition to direct antimicrobial effects, plant-derived bioactive compounds may activate plant immune responses. Plants respond to pathogen attack by activating complex defence mechanisms that limit pathogen development and stimulate the production of antimicrobial compounds and defence-related proteins. These responses may also lead to systemic resistance phenomena, such as systemic acquired resistance (SAR) or induced systemic resistance (ISR), which enhance the ability of plants to resist subsequent pathogen infections [20]. The phenylpropanoid pathway, with phenylalanine ammonia-lyase (PAL) as a key entry enzyme, plays a central role in induced resistance mechanisms, including the synthesis of phytoalexins, lignin, and other defence-related metabolites [14,21]. Activation of PAL is widely recognized as an early biochemical marker of systemic acquired resistance (SAR) and elicitor-induced defence responses in plants [20,22].

At the same time, regulatory guidelines for plant protection products emphasise the importance of evaluating phytotoxic safety and physiological effects in plants, which is critical for the practical application of natural bioactive substances [23,24]. Therefore, identifying compounds that combine direct antimicrobial activity with the ability to stimulate plant defence responses is of particular interest [18,19].

In this context, the present study aimed to evaluate cinnamon bark essential oil as a natural plant protection agent by determining its chemical profile, its antibacterial and antifungal activity against phytopathogens, its impact on bacterial metabolic activity using the MTT assay, its ability to induce plant defence responses through PAL activity, and its effects on phytotoxicity and plant growth.

## 2. Results

### 2.1. Chemical Profiling

GC–MS analysis identified 64 compounds in cinnamon bark essential oil (CBO). Components present at concentrations ≥1% are listed in Table 1, while minor constituents are provided in the Supplementary Materials (Table S1). The oil was dominated by phenylpropanoids, with cinnamaldehyde as the principal component, accompanied by eugenol and trans-cinnamyl acetate. Oxygenated monoterpenes represented a substantial fraction of the oil, particularly linalool, 1,8-cineole, and α-terpineol. Monoterpene hydrocarbons (mainly α-pinene, *p*-cymene, and γ-terpinene), as well as esters, aromatic alcohols, and the sesquiterpene β-caryophyllene, were also detected.

Overall, the chemical profile indicates the predominance of phenylpropanoids and oxygenated monoterpenes, compound groups commonly associated with strong antimicrobial activity and the modulation of plant physiological responses.

### 2.2. Microbial Assay

**Figure 1:**
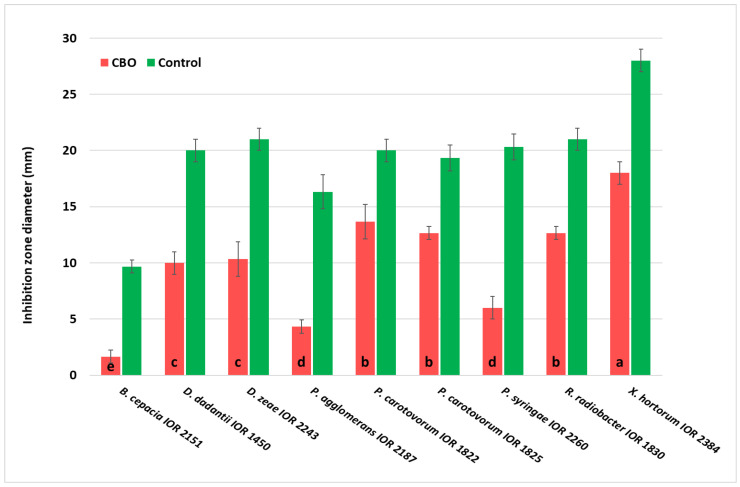
Antibacterial activity of cinnamon bark essential oil against phytopathogenic bacteria in the disk diffusion assay. Inhibition zone diameters (mm) obtained for CBO essential oil and the positive control (gentamicin) are presented. Data are expressed as mean ± SD (*n* = 3). Different letters (a–e) indicate statistically significant differences among bacterial strains treated with CBO (one-way ANOVA followed by Tukey’s post hoc test, *p* < 0.05).

Inhibition zone diameters obtained in the disk diffusion assay are presented in Figure 1. Cinnamon bark essential oil (CBO) exhibited statistically significant, strain-dependent antibacterial activity (one-way ANOVA, *p* < 0.05).

The highest susceptibility to CBO was observed in *X. hortorum* var. *carotae* IOR 2384 (approx. 18 mm), *P. carotovorum* IOR 1822, IOR 1825, and *R. radiobacter* IOR 1830 (approx. 13–14 mm). For these strains, the antibacterial effect of CBO was 60–66% of that of the positive control (gentamicin). Moderate susceptibility was observed against *Dickeya* strains, whereas lower activity was observed against *Pseudomonas syringae* IOR 2260 and *Pantoea agglomerans* IOR 2187. The lowest susceptibility was observed for *Burkholderia cepacia* IOR 2151.

For all the tested strains, the positive control produced significantly larger inhibition zones than CBO (*p* < 0.05).

The results of the antibacterial activity of cinnamon bark essential oil (CBO) against the tested phytopathogenic bacteria, including bacteriostatic (MIC), bactericidal (MBC), and metabolic effects (IC_50_), are presented in Table 2.

The minimum inhibitory concentration (MIC) values for most of the analyzed strains were 0.156 mg/mL, whereas the minimum bactericidal concentration (MBC) values were more variable, ranging from 0.156 to 1.250 mg/mL. The lowest susceptibility to the tested essential oil was observed for *B. cepacia* IOR 2151, for which the MIC and MBC values were 0.625 mg/mL and 1.250 mg/mL, respectively. For *P. agglomerans* IOR 2187, *P. syringae* IOR 2260, and *R. radiobacter* IOR 1830, bactericidal activity was detected at 0.625 mg/mL. For the remaining strains (*D. zeae* IOR 2243, *D. dadantii* IOR 1450, *X. hortorum* var. *carotae* IOR 2384, and *P. carotovorum* IOR 1822 and IOR 1825), the MIC and MBC values were identical and equal to 0.156 mg/mL, indicating a strong bactericidal effect of CBO on these pathogens.

Overall, the obtained results demonstrate strong antibacterial activity of CBO against phytopathogenic bacteria, highlighting its potential as a natural agent for the control of bacterial plant diseases.

**Figure 2:**
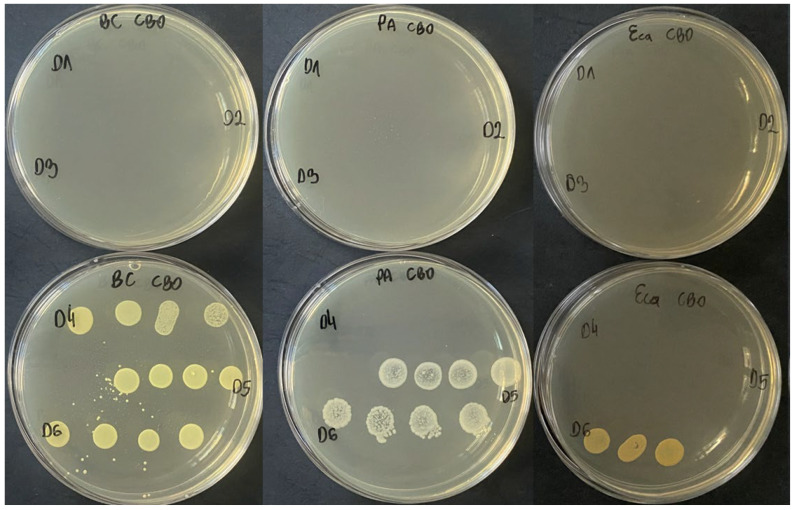
Concentration-dependent bactericidal activity of cinnamon bark essential oil (CBO) against selected phytopathogenic bacteria: BC—*B. cepacia* IOR 2151, PA—*P. agglomerans* IOR 2187, Eca—*P. carotovorum* IOR 1822. D1–D6 indicate CBO concentrations ranging from 5.0 to 0.156 mg/mL.

Figure 2 presents the concentration-dependent bactericidal effect of CBO against selected phytopathogenic bacterial strains.

IC_50_ values (Table 2), defined as the concentration of cinnamon bark essential oil (CBO) causing a 50% reduction in bacterial metabolic activity, varied among the tested phytopathogens, ranging from 0.078 to 0.410 mg/mL. The highest metabolic sensitivity to CBO was observed for strains of the genera *Dickeya* spp., *Pectobacterium* spp., and *R. radiobacter* IOR 1830. IC_50_ values for these bacteria ranged from 0.078 to 1.103 mg/mL, indicating a strong reduction in metabolic activity at relatively low oil concentrations.

Moderate metabolic susceptibility was observed for *P. agglomerans* IOR 2187 (0.181 mg/mL) and *P. syringae* IOR 2260 (0.198 mg/mL). In contrast, the highest IC_50_ values, reflecting lower metabolic sensitivity, were recorded for *X. hortorum* var. *carotae* IOR 2384 (0.300 mg/mL) and *B. cepacia* IOR 2151 (0.410 mg/mL).

These results indicate that CBO not only inhibits bacterial growth (MIC) but also reduces bacterial metabolic activity, with the magnitude of this effect being species-dependent. The low standard deviation values (SD ≤ 0.01) confirm good repeatability of the MTT assay.

The percentage inhibition of radial growth of the tested pathogens and saprotrophs is presented in Table 3. The inhibition values ranged from 42 to 100%, depending on the applied CBO concentration.

The strongest effect was observed at dose D1 (800 µg/mL), which caused nearly complete growth inhibition of all tested species (mean values close to 100%). Reducing the dose to D2 (400 µg/mL) led to decreased inhibitory effectiveness, with the mean dose effect reaching approximately 92%, although the degree of inhibition varied among species. The lowest inhibitory effect was recorded for dose D3 (200 µg/mL), for which the average growth inhibition was about 51%. Analysis of the mean pathogen effect (Y) showed that strains of *F. culmorum* (IOR Fc6, Fc12, and Fc16) exhibited the highest sensitivity to CBO, with mean inhibition levels exceeding 86%. The lowest mean inhibitory effect was observed for *R. solani* F93 (73%).

These results indicate the differential susceptibility of the tested pathogens and saprotrophs to CBO and confirm a clear dose-dependent inhibitory effect of this essential oil.

**Figure 3:**
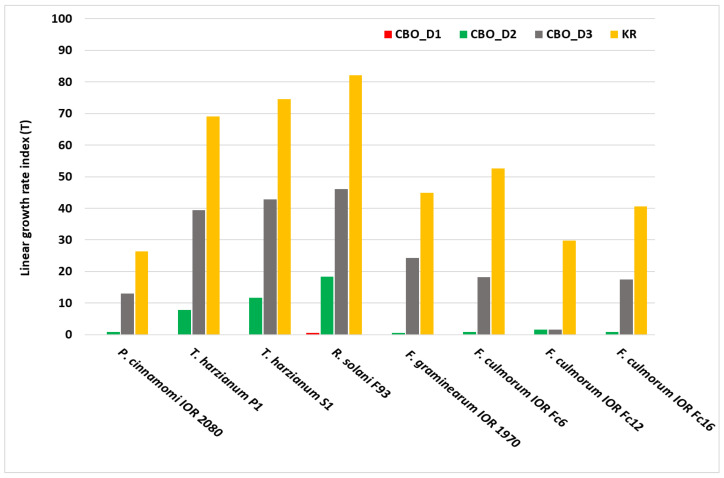
Linear growth rate index (T) of the tested organisms grown on Czapek–Dox medium supplemented with cinnamon bark essential oil (CBO). The growth rate in the untreated control (K) did not differ from that observed in the solvent control (KR); therefore, the K variant was not included in the figure. CBO doses: D1—800 µg/mL, D2—400 µg/mL, and D3—200 µg/mL; KR—negative control.

The mycelial growth rate index (T) confirmed the dose-dependent inhibitory effect of CBO (Figure 3). At the highest dose, D1 (800 µg/mL), the growth of most tested organisms was completely or nearly completely inhibited (T = 0–1), indicating a strong fungistatic effect of the oil. Reducing the dose to D2 resulted in a marked slowdown of colony growth; however, growth dynamics varied among species. The highest sensitivity to CBO was observed for strains of *Fusarium* spp., particularly Fc12 (T = 2 at D3), and *P. cinnamomi*, for which the growth rate remained clearly lower than in the control. The lowest susceptibility to CBO was recorded for *R. solani* F93 and the saprotrophic fungi *T. harzianum*, which maintained relatively high growth dynamics at the lowest dose.

These results are consistent with the data on percentage growth inhibition and indicate that CBO not only reduces the final colony size but also significantly slows the rate of mycelial development.

The percentage of inhibition or stimulation of mycelium growth after re-isolation onto oil-free medium is presented in Table 4. The results obtained indicate that prior exposure to CBO had a varied, dose-dependent effect on the ability of mycelium to regrow. The strongest effect was observed for the highest dose D1 (800 µg/mL), for which the average growth inhibition (X) was approximately 51%, significantly higher than that for the other doses (*p* < 0.05). For doses D2 and D3, the average inhibitory effect was significantly lower (~13% and ~10%, respectively). Species most affected by prior exposure were *Rhizoctonia solani* (Y = ~49%) and *Fusarium graminearum* (Y = ~43%). Moderate susceptibility was observed in *Fusarium culmorum* and *Phytophthora cinnamomi*.

Saprotrophic *T. harzianum* strains showed different responses: strain P1 exhibited growth stimulation after prior exposure (−10%), whereas S1 showed mild inhibition (15%), indicating high tolerance to CBO.

**Figure 4:**
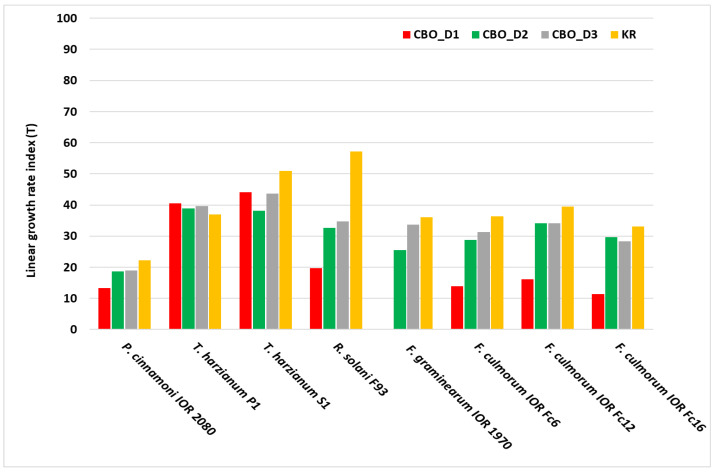
Linear growth rate index (T) of the tested organisms grown on Czapek–Dox medium (without CBO) after reisolation from CBO-treated cultures. CBO concentrations: D1—800 µg/mL, D2—400 µg/mL, and D3—200 µg/mL; KR—negative control.

The mycelial growth rate index (T) of the tested fungi and the oomycete after re-isolation onto oil-free medium is presented in Figure 4. In the control variant (KR), T values ranged from 22 to 57, depending on the species. Following prior exposure to the highest CBO dose (D1, 800 µg/mL), lowest post-treatment growth rates of phytopathogenic fungi were observed, with T values ranging from 0 to 20. A fungicidal effect was recorded for *F. graminearum* IOR 1970 (T = 0). Reducing the oil concentration to D2 and D3 resulted in a partial recovery of the growth rate, and the T values approached those of the control, indicating a gradual decline in the inhibitory effect with decreasing CBO concentration applied before reisolation. A different response was observed in saprotrophic strains of the genus *Trichoderma*. In these fungi, T values at all CBO doses (D1–D3) remained close to control levels, indicating a high tolerance to prior exposure to the essential oil.

These results show that previous exposure of mycelium to high CBO concentrations resulted in a persistent inhibitory effect on growth after transfer to oil-free medium, whereas saprotrophic *Trichoderma* strains showed a markedly greater capacity for growth regeneration.

### 2.3. Effect of Cinnamon Bark Essential Oil on Bacterial Metabolic Activity Assessed by the MTT Assay

**Figure 5:**
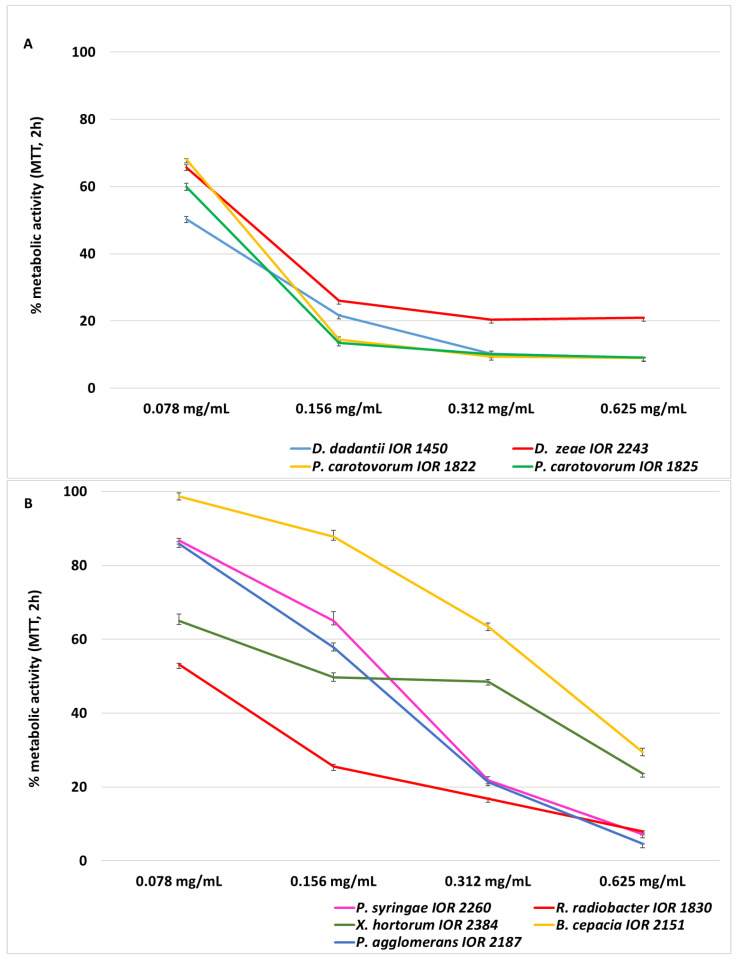
Dose-dependent effect of cinnamon bark essential oil on metabolic activity of phytopathogenic bacteria determined by the MTT assay. (**A**) Metabolic activity (% of the solvent control, KR = 100%) of *D. dadantii* IOR 1450, *D. zeae* IOR 2243, *P. carotovorum* IOR 1822 and IOR 1825 after 2 h of exposure to CBO. (**B**) Metabolic activity (% of the solvent control, KR = 100%) of *P. syringae* IOR 2260, *R. radiobacter* IOR 1830, *X. hortorum* IOR 2384, *B. cepacia* IOR 2151, and *P. agglomerans* IOR 2187 after 2 h of exposure to CBO. Values are presented as mean ± SD (*n* = 3). CBO concentrations: 0.078, 0.156, 0.312, and 0.625 mg/mL. Metabolic activity was expressed relative to the solvent control (KR = 100%); lower values indicate stronger metabolic inhibition.

The percentage of metabolic activity of bacterial cells after exposure to CBO (relative to the solvent control, KR = 100%) is presented in Figure 5A,B. The essential oil was tested at concentrations ranging from 0.625 to 0.078 mg/mL, selected based on MIC values.

CBO reduced metabolic activity in a concentration-dependent manner in all analysed strains. In isolates belonging to the family *Pectobacteriaceae* (Figure 5A), metabolic activity at concentrations of 0.156–0.625 mg/mL decreased to 9–26%, while at 0.078 mg/mL it ranged from 50% to 68%.

In the remaining strains (Figure 5B), the effect was species-dependent. At 0.625 mg/mL, metabolic activity was lowest in *P. agglomerans*, *P. syringae*, and *R. radiobacter* (5–8%), whereas higher residual activity was observed in *Xanthomonas hortorum* (24%) and *Burkholderia cepacia* (29%).

At intermediate concentrations (0.312–0.156 mg/mL), metabolic activity increased progressively, particularly in *B. cepacia*, which showed a marked rise from 29% to 63% and 88%. At 0.078 mg/mL, most strains retained >50% metabolic activity; however, interspecies differences remained evident, with *B. cepacia* (99%) and *P. syringae* (87%) showing the highest tolerance and *R. radiobacter* remaining more sensitive (53%). Overall, the MTT assay demonstrated that CBO suppressed bacterial metabolic activity in a concentration- and species-dependent manner.

### 2.4. Induction of PAL Activity in Winter Wheat by Cinnamon Bark Essential Oil

**Figure 6:**
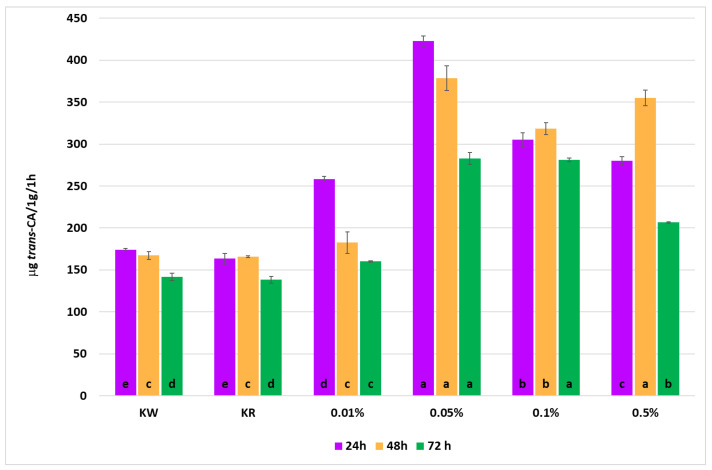
Phenylalanine ammonia-lyase (PAL) activity in leaves of *Triticum aestivum* after foliar treatment with cinnamon bark essential oil (0.01–0.5% CBO). Values are expressed as µmol *trans*-cinnamic acid h^−1^ g^−1^ fresh weight and presented as mean ± SD (*n* = 3). KW—water control; KR—solvent control. Different lowercase letters indicate significant differences among treatments (KW, KR, and CBO concentrations) within each sampling time according to Tukey’s HSD test (*p* < 0.05).

The effect of foliar application of cinnamon bark essential oil (CBO) on phenylalanine ammonia-lyase (PAL) activity in leaves of winter wheat is presented in Figure 6. PAL activity was dependent on both CBO concentration and time after application. The control variants (KW and KR) showed similar levels of enzyme activity at all sampling times, indicating no significant effect of the solvent used (0.9–1.1 µg *t*-CA g^−1^ FW h^−1^).

CBO increased PAL activity relative to the controls. The highest enzyme levels were observed 24–48 h after treatment, particularly at the 0.05% concentration, which resulted in more than a twofold increase compared with the controls. After 72 h, PAL activity declined but remained higher than in control plants. The strongest response was observed at 0.05%, with a 144% increase over the control at 24 h.

Analysis of the mean dose effect (Y) showed that the highest PAL activity occurred at 0.05% CBO, whereas the lowest values were recorded in the control variants. The mean time effect (X) indicated that PAL activity was highest at 24–48 h and decreased at 72 h after treatment.

### 2.5. Evaluation of Phytotoxic Effects and Plant Dry Mass

The highest aboveground dry mass of wheat was obtained after the application of CBO at concentrations of 0.05% (600 mg) and 0.1% (553 mg), which were significantly higher than those of the remaining variants (Table 5). The control treatments (water and solvent) did not differ from each other, whereas the 0.5% CBO treatment resulted in a slight reduction in dry mass relative to the other treatments.

The phytotoxicity index was 0 in all variants, indicating the absence of visible phytotoxic symptoms 7 days after spraying.

## 3. Discussion

The obtained results indicate that cinnamon bark essential oil is characterised by a complex chemical composition that underlies its broad biological activity. The antimicrobial effects observed in this study are consistent with the predominance of phenylpropanoids and oxygenated monoterpenes, which are known to exhibit multitarget mechanisms of action [11,12]. Cinnamaldehyde, together with eugenol and other phenylpropanoids, likely plays a major role, while oxygenated monoterpenes such as linalool and eucalyptol may enhance membrane permeability and contribute to synergistic interactions among oil constituents [11,12].

The chemical profile obtained here is in line with reports for bark oils of *Cinnamomum verum*, where cinnamaldehyde is typically the principal component [25,26,27]. However, the relatively higher proportion of oxygenated monoterpenes in the tested sample may partly explain the strong inhibitory effects observed against both bacterial and fungal phytopathogens, as these compounds are often associated with membrane disruption and interference with enzymatic systems [26].

The predominance of compounds capable of affecting membrane integrity and cellular redox balance corresponds well with the reduction in bacterial growth and metabolic activity observed in this study, suggesting that CBO acts through multiple physiological targets rather than a single specific pathway, as has been suggested in previous studies [28,29,30,31].

The results obtained in this study demonstrate that cinnamon bark essential oil exhibits strong and complex antimicrobial activity against phytopathogenic bacteria, affecting both bacterial growth and cellular metabolic activity. Previous research on the antibacterial properties of essential oils has focused mainly on human and foodborne pathogens, and most evaluations relied primarily on growth-based assays such as agar diffusion, MIC, and MBC determinations [10,11]. Notably, many of the essential oils reported to exhibit pronounced antibacterial activity are rich in phenylpropanoids, including cinnamaldehyde-, eugenol-, and anethole-dominated oils, which have been associated with membrane disruption, protein denaturation, and interference with microbial redox processes [13,16,32,33]. In particular, cinnamaldehyde has been shown to interact with bacterial cell membranes, increasing membrane permeability and causing leakage of intracellular components, while also interfering with energy metabolism and ATP synthesis in microbial cells [29,30]. This group of compounds is considered particularly effective due to their ability to penetrate lipid bilayers and disturb key physiological functions of microbial cells, which is consistent with the strong activity observed for CBO in the present study.

In contrast, data concerning phytopathogenic bacteria and metabolic-based assays remain limited. The present findings therefore extend current knowledge by showing that CBO interferes not only with cell proliferation but also with intracellular metabolic processes. The reduction in metabolic activity observed at concentrations close to or below growth-inhibitory levels suggests that cellular respiration and redox balance are among the early physiological targets of the oil’s action.

However, it should be noted that reduced MTT reduction may also result from decreased cell proliferation or reduced cell numbers rather than directly reflecting the primary mechanism of action. Therefore, the observed metabolic suppression should be interpreted with caution and considered together with growth-based parameters.

A comparison between the IC_50_ values obtained from the MTT assay and the corresponding MIC values revealed that, for some strains (e.g., *Dickeya zeae* and *Xanthomonas hortorum*), the IC_50_ values were higher than the respective MIC values. This apparent discrepancy may result from the different principles underlying the two methods. The MTT assay reflects the short-term metabolic activity of bacterial cells, whereas MIC determination evaluates the ability of microorganisms to grow and proliferate during a longer incubation period. Consequently, essential oil components may effectively inhibit bacterial growth even though some cells still retain detectable metabolic activity. It is also possible that bioactive compounds initially disrupt cellular metabolic processes without immediately preventing cell division. These observations indicate that the assessment of metabolic activity and microbial growth provides complementary information regarding the antibacterial activity and mechanisms of action of essential oils.

The species-dependent response further indicates that susceptibility to CBO is linked to physiological and structural differences among bacteria, and that the oil induces functional disturbances that may precede complete growth inhibition. These observations support the view that CBO acts through multiple cellular targets, contributing to the destabilisation of bacterial homeostasis rather than through a single, specific mechanism.

These observations underline the importance of a comprehensive approach to evaluating essential oils, combining growth-based assays with metabolic analyses, particularly in the context of their potential application as biocontrol agents in plant protection. The present results indicate that CBO exerts a multi-target effect on phytopathogenic bacteria, where metabolic disturbances appear to precede complete growth inhibition, suggesting that respiratory and redox processes may represent early physiological targets of the oil.

The pronounced reduction in metabolic activity, especially in members of the family *Pectobacteriaceae* and other sensitive strains, supports the view that CBO disrupts fundamental cellular functions in a dose-dependent manner, reinforcing its potential as a natural antimicrobial agent acting through multiple cellular pathways.

Literature data indicate that phenylpropanoid-rich essential oils, particularly those dominated by cinnamaldehyde or eugenol, frequently interfere with bacterial metabolism by disrupting membrane integrity and affecting redox-related enzyme systems. Similar metabolic suppression has been reported for plant-derived volatiles, where reductions in MTT reduction capacity reflected decreased cellular dehydrogenase activity in model Gram-negative bacteria [31,34].

The lower metabolic susceptibility observed in some strains in the present study is consistent with their generally higher tolerance to antimicrobial agents, which was also reflected in their growth-based parameters. This agreement between metabolic and MIC/MBC responses supports the usefulness of metabolic assays as an early indicator of antibacterial activity.

Because the MTT assay reflects overall dehydrogenase activity, a decrease in this signal likely precedes complete growth inhibition, suggesting that essential oils may disrupt respiratory and redox processes before exerting full bacteriostatic or bactericidal effects. Although MTT-based approaches are less frequently applied than classical MIC/MBC determinations, particularly in phytopathogen research, previous studies have shown that this method provides sensitive information on physiological disturbances induced by essential oils [35,36].

Therefore, combining growth-based and metabolic assays offers a more comprehensive picture of antibacterial activity, especially when evaluating natural products with multi-target mechanisms of action.

Previous studies on cinnamon bark essential oil have focused mainly on opportunistic fungi, such as *Candida* spp., *Aspergillus* spp. and *Penicillium* spp., where strong in vitro antifungal activity has been reported [37,38,39]. This activity is commonly attributed to cinnamaldehyde and other phenylpropanoids and has been described as fungistatic or fungicidal depending on concentration and exposure conditions [26,37,38,40].

In the present study, CBO markedly inhibited mycelial growth of several phytopathogenic fungi in the poisoned-medium assay. The lack of a positive control in the antifungal inhibition assay constitutes a limitation of this study; therefore, the obtained results should be interpreted as a relative measure of the antifungal activity of the tested essential oil rather than a direct comparison with conventional antifungal agents. Importantly, the inhibitory effect persisted after transfer to oil-free medium in selected species, indicating irreversible cellular damage rather than only transient growth arrest. Such reisolation-based assessment, still rarely included in antifungal studies, provides valuable information on the durability of antifungal action and supports the occurrence of fungicidal effects under the tested conditions [41,42].

A further noteworthy observation was the selective activity of CBO. While phytopathogenic fungi showed high susceptibility, saprotrophic *Trichoderma* strains exhibited markedly greater tolerance and, in some cases, even enhanced growth after prior exposure. This differential response suggests that CBO may preferentially suppress plant pathogens while exerting limited pressure on beneficial soil fungi, a property desirable for plant protection applications [41,43,44,45].

Overall, these findings suggest that CBO interferes not only with active mycelial growth but also with post-exposure recovery processes, indicating long-lasting cellular damage and highlighting its potential as a selective antifungal component for plant protection formulations.

The results indicate that foliar application of cinnamon bark essential oil (CBO) activated defence responses in winter wheat, as reflected by increased PAL activity. Because PAL is a key entry enzyme of the phenylpropanoid pathway, its stimulation suggests activation of phenylpropanoid-related defence metabolism. Although PAL activation is widely recognised as a general marker of induced resistance and elicitor action, reports specifically addressing the elicitor activity of CBO on PAL remain scarce, making the present findings particularly relevant in this context [14,46]. It should be noted that no reference elicitor was included in the PAL activity assay; therefore, the observed increase in PAL activity should be interpreted evidence of elicitor-like activity rather than a direct comparison with established elicitors.

The transient character of the response, with peak activity during the first two days after treatment, is consistent with early defence reactions to elicitors described in plants, suggesting that CBO may function as an elicitor-type stimulus rather than inducing prolonged physiological disturbance [14,19,46,47].

Importantly, this biochemical activation was not associated with phytotoxic effects, and moderate CBO concentrations were accompanied by improved shoot biomass. This combination of defence-related enzyme activation and absence of growth suppression indicates that CBO may exhibit plant defence-stimulating properties without visible negative effects on plant growth.

Overall, these findings indicate that CBO treatment was associated with defence-related enzyme activation while maintaining normal growth, which is relevant in the context of developing plant protection-supporting formulations. Similar stimulation of defence-related enzymes by plant essential oils has been reported in other plant systems [8,48].

## 4. Materials and Methods

### 4.1. Chemical Composition of Cinnamon Bark Essential Oil

Essential oil distilled from the bark of *Cinnamomum verum* J. Presl (syn. *Cinnamomum zeylanicum* Blume, Lauraceae) was purchased from Herbiness Sp. z o.o., Chomiec, Poland. According to the manufacturer’s declaration, the essential oil was obtained by steam distillation of the bark of *Cinnamomum verum* branches originating from Sri Lanka (Hambantota District). Chemical analysis was performed using a Varian CP-3800 gas chromatograph coupled with a Saturn 2000 mass spectrometer (Varian, Walnut Creek, CA, USA) equipped with a Zebron ZB-5 MSi column (30 m × 0.25 mm × 0.25 µm; Phenomenex, Torrance, CA, USA). The temperature programme was as follows: the initial oven temperature was 60 °C, increased at 2 °C·min^−1^ to 200 °C and subsequently increased at 10 °C·min^−1^ to 290 °C. Mass spectra were recorded at a scanning rate of 1 scan·s^−1^ in the range of 38–300 *m*/*z* using electron impact (EI) ionisation at 70 eV. Samples were injected at a split ratio of 1:50, and helium gas was used as the carrier gas at a flow rate of 1.0 mL·min^−1^. Additionally, chemical ionisation (CI) mode was applied to determine the molecular masses of individual components. Methanol was used as a reagent gas with CI storage level set at 19 *m*/*z* and ejection amplitude 15 V. During the analysis signals were collected with a scan time of 870 ms. Chromatograms, the complete table of identified components, and the source file are available in Supplementary Materials (Figures S1 and S2).

The following methods were used to identify the CBO components: (i) comparison of obtained mass spectra with databases NIST 23 (National Institute of Standards and Technology) and FFNSC (Mass Spectra of Flavors and Fragrances of Natural and Synthetic Compounds); (ii) comparison of calculated linear retention indices (LRIs) using a retention index calculator with values presented in NIST 23 and FFNSC; (iii) comparison of retention times of unknown compounds with authentic standards when available. Linear retention indices were calculated relative to a homologous series of n-alkanes analysed under identical chromatographic conditions. Quantitative analysis was based on response factors calculated for groups of CBO constituents. Following software was used for the analysis: Varian MS Workstation v. 6.5 (Varian, Walnut Creek, CA, USA), AMDIS (Automated Mass Spectral Deconvolution and Identification System), ver. 2.73; National Institute of Standards and Technology, Gaithersburg, MD, USA and ACD/Spectrus Processor v. 2021.2.1 (Advanced Chemistry Development, Inc., Toronto, ON, Canada). An individual Excel macro (Supplementary Materials Figure S2) was used for the calculation of retention indices (RIs) [49,50].

### 4.2. Antimicrobial Assay

#### 4.2.1. Test Microorganisms

The antimicrobial activity of cinnamon bark essential oil was evaluated against a selected panel of plant-associated microorganisms, including Gram-negative phytopathogenic bacteria, filamentous fungi, and one oomycete. Gram-negative rods comprised: *Burkholderia cepacia* IOR 2151, *Dickeya zeae* IOR 2243, *Dickeya dadantii* IOR 1450 (pv. *Erwinia chrysanthemi*), *Pectobacterium carotovorum* IOR 1822 and 1825 (pv. *Erwinia carotovora*), *Pantoea agglomerans* IOR 2187, *Pseudomonas syringae* IOR 2260, *Agrobacterium radiobacter* IOR 1830 (pv. *Rhizobium radiobacter*); *Xanthomonas hortorum* var. *carotae* IOR 2384; Filamentous fungi and fungus-like organisms comprised: *Fusarium culmorum* IOR strains Fc5 (79), F6 (1596), Fc12 (8), *F. culmorum* Fc16, *Fusarium graminearum* IOR strain 1970, *Rhizoctonia solani* F93 and saprotrophic fungi *Trichoderma harzianum* ThP1, ThS1 and the oomycete *Phytophthora cinnamomi* IOR 2080.

Bacterial strains originated from the Culture Collection of Plant Pathogens, Institute of Plant Protection, National Research Institute (IOR, Poznań, Poland). Fungal and oomycete cultures were obtained either from IOR or from the authors’ collection. All strains were routinely maintained on agar slants at 4 °C and subcultured before to experiments. Before antimicrobial assays, bacterial cultures were freshly grown for 24 h on Luria–Bertani agar (LBA; Sigma-Aldrich, St. Louis, MO, USA), while fungi and the oomycete were grown on potato dextrose agar (PDA; Sigma-Aldrich) for 5–7 days at 25 ± 1 °C in the dark to obtain actively growing margins.

#### 4.2.2. Agar Disc Diffusion Assay

The antibacterial activity of cinnamon bark oil (CBO) was evaluated using the agar disk diffusion assay according to a previously described protocol [51,52], with modifications concerning the solvent system. Bacterial suspensions were adjusted to 0.5 McFarland standard (≈1.5 × 10^8^ CFU/mL) verified spectrophotometrically at 600 nm (spectrophotometer VIS-723G, Rayleigh, Beijing, China).

A volume of 100 µL of bacterial suspension was evenly spread onto Luria–Bertani agar (LBA) plates (agar depth 4.0 ± 0.5 mm). Sterile paper discs (Whatman No. 1, 6 mm diameter) were impregnated with 30 µL of CBO solution, corresponding to 500 µg of essential oil per disc. The selected dose of essential oil (500 µg/disc) was applied as a preliminary screening concentration to enable the initial evaluation of antibacterial activity prior to further quantitative determination of MIC values. CBO was dissolved in 10% (*v*/*v*) DMSO containing 0.05% (*v*/*v*) Tween 80 to enhance solubilization and dispersion. The final concentration of DMSO in the assay did not exceed 1% (*v*/*v*), and solvent control discs were included to exclude any potential effect of DMSO on bacterial membrane permeability. Discs were dried under sterile airflow for 10 min before placement. Negative control discs contained solvent only, while positive controls contained gentamicin (100 µg/disc). Plates were incubated at 28 °C for 24 h. Inhibition zones were measured with a digital caliper, and the disc diameter was subtracted. Experiments were performed in triplicate and results were expressed as mean ± standard deviation (SD). Zone diameters were expressed in millimeters.

#### 4.2.3. Determination of Minimum Inhibitory Concentration (MIC) and Minimum Bactericidal Concentration (MBC)

MIC and MBC values of cinnamon bark essential oil (CBO) were determined using the broth microdilution method in 96-well microtiter plates, according to a previously described protocol [52,53], with modifications in the concentration range and control system. Bacterial strains were cultured on Luria–Bertani agar (LBA). Bacterial suspensions were adjusted to a 0.5 McFarland standard (≈1.5 × 10^8^ CFU/mL) and diluted to obtain a final density of approximately 5 × 10^5^ CFU/mL in each well.

CBO was dissolved in DMSO containing Tween 80 (final concentrations ≤1% and ≤0.05%, respectively) and serially diluted in Luria–Bertani broth to final concentrations of 5.0–0.156 mg/mL. Growth control (medium + inoculum), solvent control, and sterile medium blank were included. The final volume in each well was 200 µL.

Plates were incubated at 28 °C for 24 h. Optical density at 600 nm was measured using a microplate reader. MIC was defined as the lowest concentration showing no visible growth and no increase in OD relative to the blank.

MBC was determined by plating 20 µL aliquots from growth-inhibited wells onto Luria–Bertani agar and incubating for 24 h at 28 °C. MBC was defined as the lowest concentration resulting in no colony growth.

#### 4.2.4. MTT Assay

Metabolic activity of bacteria after exposure to cinnamon bark essential oil (CBO) was assessed using the tetrazolium salt reduction assay (MTT), adapted from previously described bacterial viability protocols [54,55].

Bacterial cells in the exponential growth phase were adjusted to OD_600_ ≈ 0.5 and incubated with CBO at concentrations of 0.628–0.078 mg/mL. CBO was dissolved in DMSO with Tween 80 (final concentrations ≤1% and ≤0.05%, respectively).

After 2 h of incubation at 28 °C, cells were centrifuged (6000× *g*, 5 min), and the cell pellets were washed twice with PBS to remove residual CBO prior to MTT addition, minimizing the possibility of non-enzymatic reduction of the tetrazolium salt. Cells were resuspended in LB medium, and MTT solution was added to a final concentration of 0.5 mg/mL. Samples were incubated for 2 h at 28 °C in the dark. Formazan crystals were dissolved in isopropanol and absorbance was measured at 570 nm. Metabolic activity (%) was expressed as expressed relative to the solvent-treated growth control. IC_50_ was defined as the CBO concentration causing a 50% reduction in metabolic activity and was calculated by logarithmic interpolation.

#### 4.2.5. Antifungal Activity

Antifungal activity of cinnamon bark essential oil was evaluated using the Poisoned Food Technique [56] as previously described [51], with minor modifications. CBO dissolved in 10% (*v*/*v*) DMSO, was incorporated into molten Czapek–Dox agar (cooled to 50–55 °C) to obtain final concentrations of 800, 400, and 200 µg/mL (D1–D3). Control plates contained the solvent only. Each plate was inoculated with two mycelial plugs (5 mm diameter) cut from the actively growing margin of a 7-day-old fungal colony and placed opposite each other on the agar surface. Plates were incubated under species-specific conditions, and radial colony growth was recorded daily until control colonies reached the plate edge. Experiments were performed in four replicates. To evaluate fungicidal activity, mycelial plugs from growth-inhibited plates were transferred onto fresh Czapek–Dox agar without CBO and incubated to assess regrowth. Growth parameters, including the linear growth index (T) and the coefficient of radial growth inhibition, were calculated as described previously [52].

### 4.3. Induction of Plant Defence Mechanisms by Cinnamon Bark Essential Oil—Evaluation of Elicitor Potential

The experiment aimed to evaluate the effect of cinnamon bark essential oil on the induction of plant defence responses, with particular emphasis on phenylalanine ammonia-lyase (PAL) activity. The study was conducted on 7-day-old seedlings of winter wheat (*Triticum aestivum* L.). Plants were grown in a phytotron under a 16 h light/8 h dark photoperiod at temperatures of 26/18 °C (day/night). Seedlings were cultivated in a soil–sand mixture (2:1, *v*/*v*; pH 6.0–6.5). CBO was emulsified in distilled water containing Tween 80 (0.05%, *v*/*v*). Plants were sprayed once with CBO solutions at concentrations of 0.01%, 0.05%, 0.1%, and 0.5% (*v*/*v*), applying 2 mL per pot. Distilled water served as the negative control (Kw), whereas a solution containing Tween 80 (0.05%) without CBO was used as the solvent control (K0). No reference elicitor was included in the PAL activity assay. Each treatment was performed in three biological replicates (three pots per treatment).

#### 4.3.1. Determination of Phenylalanine Ammonia-Lyase (PAL) Activity

Leaf samples (300 mg fresh weight) were collected from each pot separately at 0, 24, 48, and 72 h after CBO application (*n* = 3). The material was immediately frozen in liquid nitrogen and stored at −70 °C until analysis. PAL activity was determined according to a modified method of Peltonen and Karjalainen [57]. Leaf tissue was homogenized in 4 mL of extraction buffer containing 50 mM Tris–HCl (pH 8.5), 14.4 mM 2-mercaptoethanol, and 5% (*w*/*v*) polyvinylpolypyrrolidone (PVPP), with the addition of quartz sand. Homogenates were centrifuged twice (10 min and 7 min, 13,000× *g*, 4 °C).

The reaction mixture consisted of 0.5 mL of the enzyme extract (supernatant) and 2.5 mL of 0.2% L-phenylalanine prepared in 50 mM Tris–HCl buffer (pH 8.5). D-phenylalanine was used in the control reaction. Samples were incubated for 20 h at 30 °C. The reaction was terminated by adding 0.5 mL of 35% (*v*/*v*) trifluoroacetic acid (TFA), followed by centrifugation. Absorbance was measured at 290 nm using a Cintra 40 UV–VIS spectrophotometer (GBC Scientific Equipment, Australia). PAL activity was expressed as µmol of *trans*-cinnamic acid (*t*-CA) produced per hour per gram of fresh weight (µmol *t*-CA h^−1^ g^−1^ FW). All reagents were purchased from Merck (Darmstadt, Germany), unless stated otherwise.

#### 4.3.2. Phytotoxicity Assessment and Dry Mass Determination

Phytotoxic effects were evaluated 7 days after spraying using a phytotoxicity index (using a 0–4 scale) as described previously [52]. Above-ground dry mass was determined by drying plant material at 50 °C for 24 h, followed by additional drying at 105 °C for 1 h to obtain constant weight. Plants were weighed in ten biological replicates. Results were expressed in milligrams (mg) and as a percentage of the solvent control (K0 = 100%).

### 4.4. Statistical Analysis

Statistical analyses were performed using one-way or two-way ANOVA, depending on the experiment, followed by Tukey’s HSD post hoc test. Differences were considered statistically significant at *p* < 0.05. Normality and homogeneity of variance were verified using the Shapiro–Wilk and Levene tests. Analyses were conducted using Statistica version 13 (TIBCO Software Inc., Palo Alto, CA, USA).

## 5. Conclusions

The results demonstrate that cinnamon bark essential oil, characterised by a composition rich in cinnamaldehyde and oxygenated monoterpenes, exhibits pronounced biological activity against phytopathogens. CBO inhibited the growth and metabolic activity of selected phytopathogenic bacteria, as well as fungi and oomycetes, suggesting that the oil may affect multiple cellular targets contributing to its antimicrobial activity. The persistence of inhibitory effects in some fungal pathogens indicates that, beyond temporary growth suppression, the oil may induce longer-term physiological disturbances in microbial cells.

In plant experiments, foliar application of CBO induced PAL activity in winter wheat without visible phytotoxic effects, and moderate concentrations were associated with increased shoot biomass. This indicates that CBO may act not only as a direct antimicrobial agent but also as a plant defence elicitor with mild biostimulatory potential.

Overall, these findings support the potential use of cinnamon bark essential oil as a multifunctional natural agent combining antimicrobial efficacy with plant defence stimulation, which may be valuable in sustainable crop protection strategies.

## Figures and Tables

**Table 1:** Chemical profile of cinnamon bark oil (CBO).

No.	Compounds	RT (min)	Contribution [%]
1	Cinnamaldehyde	24.6	41.3
2	Linalool	14.0	18.3
3	Eucalyptol	10.6	6.8
4	Eugenol	29.3	4.0
5	*trans*-Cinnamyl acetate	35.1	3.3
6	Caryophyllene	33.2	3.3
7	α-Terpineol	19.6	3.2
8	α-Pinene	6.6	2.0
9	Isobornyl acetate	25.4	1.7
10	*p*-Cymene	10.2	1.4
11	Benzaldehyde	7.7	1.3
12	β-Phenethyl alcohol	14.7	1.3
13	α-Terpinyl acetate	28.9	1.3
14	γ-Terpinene	11.8	1.2
15	Benzyl benzoate	52.9	1.2
16	β-Phenethyl acetate	23.2	1.1

RT—Retention time obtained on a ZB-5 MSi column under the chromatographic conditions described in Section 4.1.

**Table 2:** Bacteriostatic (MIC), bactericidal (MBC), and concentration causing 50% reduction in metabolic activity (IC_50_) of cinnamon bark essential oil against tested phytopathogenic bacteria (mg/mL).

Tested Pathogens	MIC	MBC	IC_50_ (MTT)
*Burkholderia cepacia* IOR 2151	0.625	1.250	0.410 ± 0.01 c
*Dickeya dadantii* IOR 1450	0.156	0.156	0.078 ± 0.00 a
*Dickeya zeae* IOR 2243	0.156	0.156	1.103 ± 0.01 a
*Pantoea agglomerans* IOR 2187	0.312	0.625	0.181 ± 0.00 b
*Pectobacterium carotovorum* IOR 1822	0.156	0.156	0.098 ± 0.00 a
*Pectobacterium carotovorum* IOR 1825	0.156	0.156	0.095 ± 0.00 a
*Pseudomonas syringae* IOR 2260	0.156	0.625	0.198 ± 0.00 b
*Rhizobium radiobacter* IOR 1830	0.312	0.625	0.084 ± 0.00 a
*Xanthomonas hortorum* var. *carotae* IOR 2384	0.156	0.156	0.300 ± 0.00 c

MIC—Minimum Inhibitory Concentration; MBC—Minimum Bactericidal Concentration; IC_50_—concentration causing a 50% reduction in metabolic activity determined using the MTT assay. IC_50_ values are presented as mean ± SD (*n* = 3). Different letters indicate statistically significant differences among bacterial strains (one-way ANOVA followed by Tukey’s post hoc test, *p* < 0.05). MIC and MBC values were identical across replicates; therefore, SD = 0 and statistical analysis was not applicable.

**Table 3:** Percentage inhibition of mycelial growth (H, %) of saprotrophic and polyphagous phytopathogenic fungi and the oomycete *Phytophthora cinnamomi* grown on Czapek–Dox medium supplemented with cinnamon bark essential oil (CBO).

Tested Strains	KR (mm)	D1 (%)	D2 (%)	D3 (%)	Y
*Phytophthora cinnamomi* IOR 2080	24.3 ± 0.96	100.0 ± 0.00 a	96.9 ± 3.95 a	48.5 ± 2.38 abc	81.8 ABC
*Trichoderma harzianum* P1	63.3 ± 0.96	100.0 ± 0.00 a	88.9 ± 10.25 ab	42.7 ± 5.06 c	77.2 BCD
*Trichoderma harzianum* S1	70.0 ± 0.00	100.0 ± 0.00 a	84.6 ± 5.99 ab	42.5 ± 1.37 c	75.7 CD
*Rhizoctonia solani* F93	75.0 ± 5.77	99.3 ± 0.77 a	77.3 ± 15.28 b	43.0 ± 2.28 c	73.2 D
*Fusarium graminearum* IOR 1970	41.5 ± 0.58	100.0 ± 0.00 a	98.8 ± 2.41 a	47.6 ± 5.34 ab	82.1 ABC
*Fusarium culmorum* IOR Fc6	48.3 ± 0.96	100.0 ± 0.00 a	98.4 ± 1.04 a	65.3 ± 8.35 a	87.9 A
*Fusarium culmorum* IOR Fc12	27.8 ± 0.96	100.0 ± 0.00 a	94.6 ± 2.08 a	63.1 ± 3.45 ab	85.9 A
*Fusarium culmorum* IOR Fc16	37.0 ± 4.62	100.0 ± 0.00 a	98.0 ± 1.35 a	56.1 ± 20.63 abc	84.7 AB
X	—	99.9 A	92.2 B	51.1 C	—

Growth inhibition was calculated as the percentage reduction in colony diameter relative to the negative control (KR). Data are presented as mean ± SD. CBO concentrations: D1—800 µg/mL, D2—400 µg/mL, D3—200 µg/mL. X—mean antifungal effect for a given CBO dose; Y—mean susceptibility of a given fungal species. Mean values followed by the same lowercase or uppercase letters within columns do not differ significantly according to Tukey’s HSD test (*p* < 0.05).

**Table 4:** Percentage inhibition or stimulation of mycelial growth (H/S, %) of saprotrophic and polyphagous phytopathogenic fungi and the oomycete *P. cinnamomi* grown on Czapek–Dox medium (without CBO) after reisolation from CBO-treated cultures.

Tested Strains	KR (mm)	D1 (%)	D2 (%)	D3 (%)	Y
*P. cinnamomi* IOR 2080	23.50 ± 0.58	47.55 ± 28.7 bc	12.77 ± 2.4 b	10.64 ± 3.5 b	21.9 C
*T. harzianum* P1	38.50 ± 0.58	−7.8 ± 1.5 d	−12.3 ± 3.3 d	−11.04 ± 4.4 d	−10.4 E
*T. harzianum* S1	54.00 ± 0.82	16.7 ± 0.2 cd	14.3 ± 2.8 b	14.35 ± 6.7 b	15.1 D
*R. solani* F93	73.50 ± 2.38	69.8 ± 24.5 ab	39.9 ± 0.8 a	38.93 ± 0.8 a	49.4 A
*F. graminearum* IOR 1970	37.50 ± 0.58	100.0 ± 0.0 a	22.7 ± 3.8 ab	5.33 ± 1.5 c	42.6 AB
*F. culmorum* IOR Fc6	36.75 ± 0.59	63.3 ± 14.8 ab	15.0 ± 1.5 b	10.88 ± 4.1 b	29.7 BC
*F. culmorum* IOR Fc12	39.25 ± 1.26	60.5 ± 4.4 b	8.3 ± 2.1 bc	4.46 ± 3.3 c	24.4 C
*F. culmorum* IOR Fc16	33.75 ± 0.96	64.5 ± 21.1 ab	5.19 ± 1.6 c	8.15 ± 2.4 b	25.9 BC
X	—	51.1 A	13.2 B	10.21 B	—

Growth inhibition or stimulation was calculated as the percentage reduction or increase in colony diameter relative to the negative control (KR). Data are presented as mean ± SD. CBO concentrations: D1—800 µg/mL, D2—400 µg/mL, D3—200 µg/mL. X—mean antifungal effect for a given CBO dose; Y—mean susceptibility of a given fungal species. Mean values followed by the same lowercase or uppercase letters within columns do not differ significantly according to Tukey’s HSD test (*p* < 0.05).

**Table 5:** Plant dry mass (mg) and phytotoxic effect 7 days after foliar application of cinnamon bark essential oil (CBO).

Treatment	Dry Mass (mg)	Phytotoxic Effect (Scale 0–4)
KW	527.33 ± 6.03 c	0
KR—solvent control	527.33 ± 8.55 c	0
CBO_0.01%	525.33 ± 12.50 c (99.6%)	0
CBO_0.05%	600.33 ± 10.41 a (113.9%)	0
CBO_0.1%	552.67 ± 5.03 b (104.8%)	0
CBO_0.5%	505.00 ± 8.54 c (95.8%)	0

KW—water control; KR—solvent control. Values in parentheses represent percentage relative to the solvent control (KR = 100%). Data were analysed using one-way ANOVA followed by Tukey’s post hoc test (*p* < 0.05). Different letters indicate significant differences among treatments.

## Data Availability

Data will be made available on request.

## Supplementary Materials

The following supporting information can be downloaded at: https://www.mdpi.com/article/10.3390/molecules31061036/s1, Figure S1. EI GC-MS chroatogram of *Cinnamomum verum* J. Presl bark essential oil. Figure S2. CI (methanol) GC-MS chroatogram of *Cinnamomum verum* J. Presl bark essential oil. Figure S3. Comparison of CI and EI chromatograms of *Cinnamomum verum* J. Presl bark essential oil. Table S1. Full characterization of *Cinnamomum verum* J. Presl bark essential oil. Figure S4. EI (left) and CI (right) MS spectra of unknown, time 42.06 min compound. Figure S5. EI (left) and CI (right) MS spectra of unknown, time 72.28 min compounds.

## Abbreviations

The following abbreviations are used in this manuscript:

CBO	cinnamon bark essential oil
CDA	Czapek–Dox agar medium
DMSO	dimethyl sulfoxide
IC_50_	half maximal inhibitory concentration
LB/LBA	Luria–Bertani broth/Luria–Bertani agar
MBC	minimum bactericidal concentration
MIC	minimum inhibitory concentration
MTT	3-(4,5-dimethylthiazol-2-yl)-2,5-diphenyltetrazolium bromide assay
PAL	phenylalanine ammonia-lyase
PBS	phosphate-buffered saline
