# Supplementary material for: Cinnamon Bark Essential Oil as a Natural Plant Protection Agent: Chemical Profile, Antimicrobial Activity, and Defence Induction
Source: Molecules. 2026 Mar 20;31(6):1036. doi: 10.3390/molecules31061036 (PMC13028868; doi:10.3390/molecules31061036)
Supplement: Supplementary file 1 [file molecules-31-01036-s001.zip › molecules-4189131-supplementary.pdf]

Suplementarny materials:

Figure S1. EI GC-MS chroatogram of *Cinnamomum verum* J. Presl bark essential oil

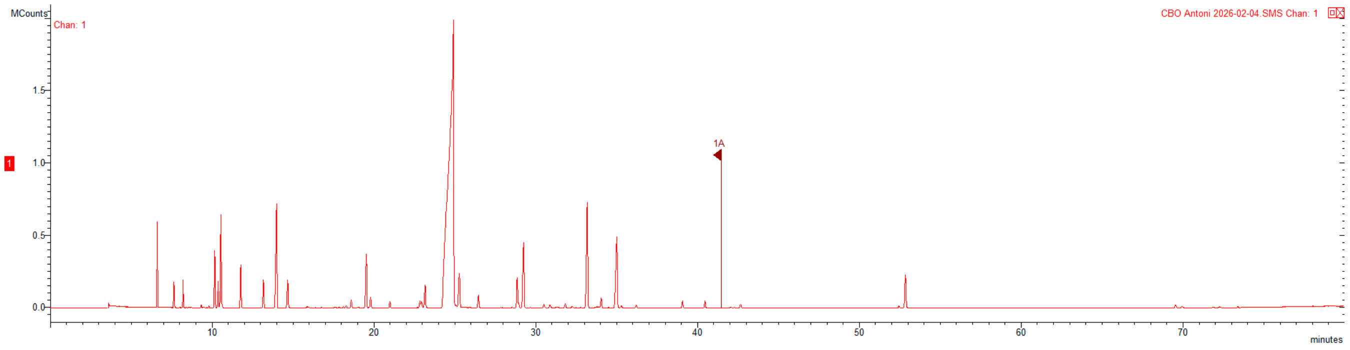

FigureS2. CI (methanol) GC-MS chroatogram of *Cinnamomum verum* J. Presl bark essential oil

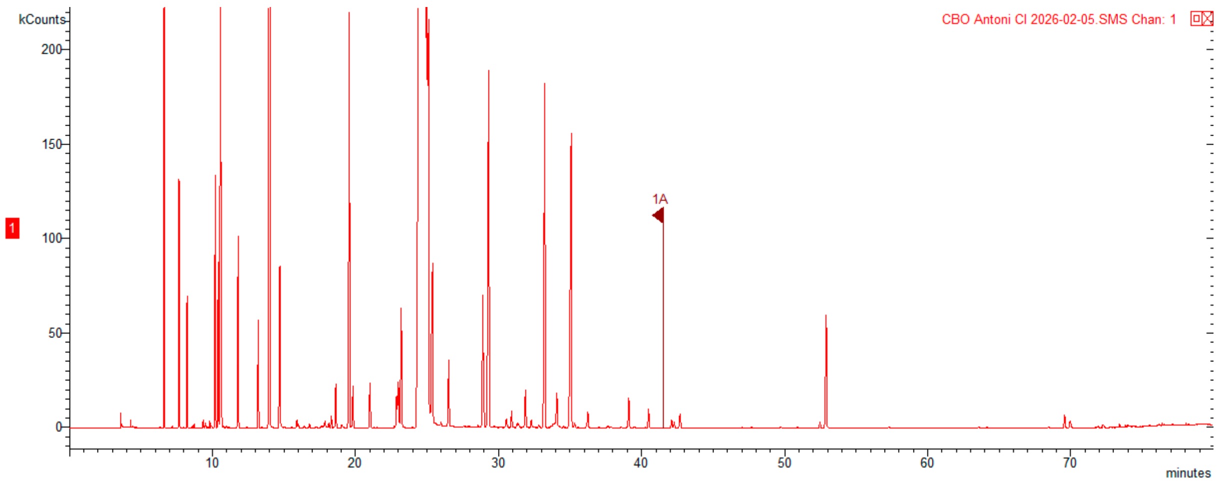

FigureS3. Comparison of CI and EI chromatograms of *Cinnamomum verum* J. Presl bark essential oil

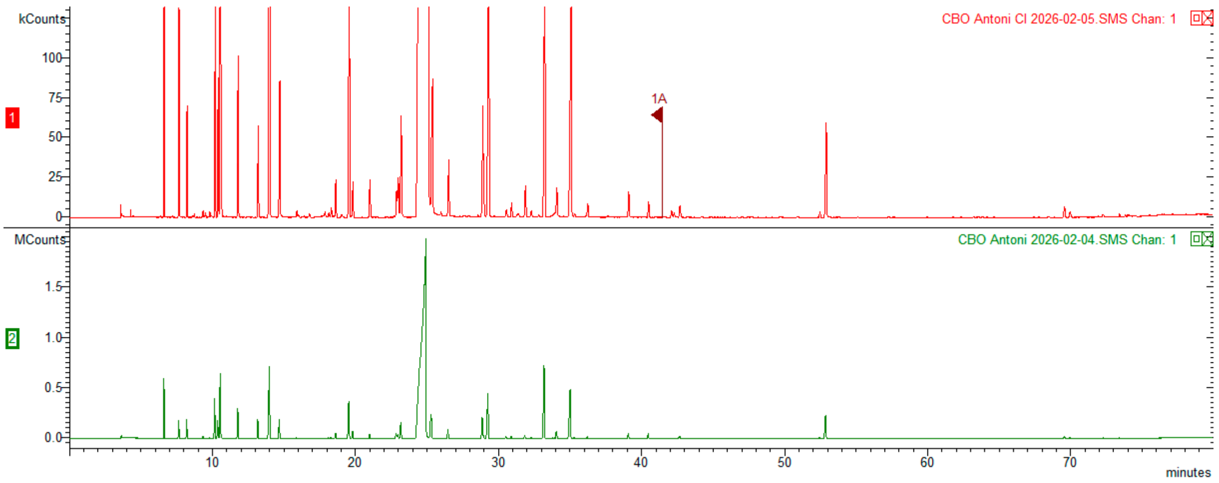

Table S1. Full characterization of *Cinnamomum verum* J. Presl bark essential oil

No.	Peak Name	tR (min)	KI exp.	KI lit.	Area (%)
1	α-Pinene	6.635	**941**	937	1.991
2	Camphene	7.207	**958**	952	0.013
3	Benzaldehyde	7.665	**971**	962	1.333
4	β-Pinene	8.236	**987**	979	0.665
5	β-Myrcene	8.579	**997**	991	0.008
6	Sulcatol	8.737	**1002**	993	0.021
7	α-Phellandrene	9.367	**1014**	1005	0.05
8	Isobutyric acid, isopentyl ester	9.538	**1017**	1014	0.04
9	α-Terpinene	9.824	**1023**	1017	0.037
10	p-Cymene	10.195	**1031**	1025	1.446
11	Limonene	10.407	**1035**	1030	0.94
12	Eucalyptol	10.563	**1038**	1032	6.758
13	1-Propanol, 2-(2-hydroxypropoxy)-	10.963	**1046**	1046	0.014
14	γ-Terpinene	11.807	**1064**	1060	1.175
15	Terpinolene	13.195	**1092**	1088	0.721
16	Linalool	14.026	**1103**	1100	18.293
17	β-Phenethyl alcohol	14.71	**1119**	1116	1.303
18	Dihydrolinalool	15.898	**1139**	1134	0.045
19	1-Terpinenol	15.998	**1141**	1137	0.015
20	trans-dihydro-α-Terpineol	16.414	**1148**	1147	0.014
21	β-Terpineol	16.786	**1154**	1153	0.039
22	Benzoic acid	17.617	**1169**	1172	0.012
23	Ethyl benzoate	17.875	**1173**	1173	0.055
24	linalool oxide D	18.133	**1177**	1175	0.039
25	β-Phenethyl formate	18.318	**1181**	1178	0.088
26	Terpinen-4-ol	18.629	**1186**	1183	0.331
27	p-Cymen-8-ol	19.058	**1193**	1185	0.044
28	α-Terpineol	19.574	**1193**	1189	3.196
29	γ-Terpineol	19.829	**1204**	1200	0.315
30	(Z)-Cinnamaldehyde	21.015	**1225**	1220	0.396
31	Linalyl acetate	22.875	**1255**	1254	0.125
32	Geraniol	22.988	**1257**	1255	0.262
33	β-Phenethyl acetate	23.201	**1260**	1258	1.057
34	Cinnamaldehyde	24.571	**1282**	1276	**41.307**
35	Isobornyl acetate	25.387	**1295**	1287	1.692
36	Geranyl formate	25.978	**1305**	1300	0.034
37	2-Propen-1-ol, 3-phenyl-	26.52	**1313**	1311	0.593
38	(1-Methylpenta-2,4-dienyl)benzene	28.582	**1347**	1348	0.018
39	α-Terpinyl acetate	28.897	**1352**	1350	1.296
40	Eugenol	29.32	**1358**	1357	4.004

41	Methyl benzyl butyrate	29.706	**1365**	1364	0.014
42	Copaene	30.551	**1378**	1376	0.099
43	Neryl acetate	30.909	**1384**	1382	0.147
44	Benzene, 1,3-hexadienyl-	31.367	**1391**	1385	0.085
45	Vanillin	31.881	**1400**	1403	0.358
46	Isocaryophyllene	32.294	**1406**	1407	0.087
47	Funebrene	32.822	**1415**	1414	0.034
48	Caryophyllene	33.222	**1422**	1419	3.284
49	α-Bergamotene	34.078	**1436**	1435	0.322
50	trans-Cinnamyl acetate	35.078	**1453**	1446	3.328
51	Humulene	35.32	**1457**	1454	0.037
52	Cinnamic acid, ethyl ester	36.236	**1472**	1466	0.162
53	α-Selinene	37.652	**1495**	1497	0.023
54	Acetyleugenol	39.112	**1521**	1524	0.274
55	δ-Cuprenene	40.486	**1544**	1544	0.166
56	Unknown 1	42.088	**1572**	n.d.	0.07
57	γ-Undecanolide	42.286	**1576**	1576	0.042
58	Caryophyllene oxide	42.688	**1583**	1581	0.142
59	Cedryl acetate	52.472	**1763**	1763	0.063
60	Benzyl Benzoate	52.898	**1771**	1764	1.164
61	Benzoic acid, phenethyl ester	57.296	**1857**	1856	0.015
62	Methyl octadecanoate	69.56	**2124**	2125	0.132
63	Oleic acid	69.961	**2136**	2142	0.082
64	Unknown 2	72.282	**2206**	n.d.	0.037

Figure S4. EI (left) and CI (right) MS spectra of unknown, time 42.06 min compound:

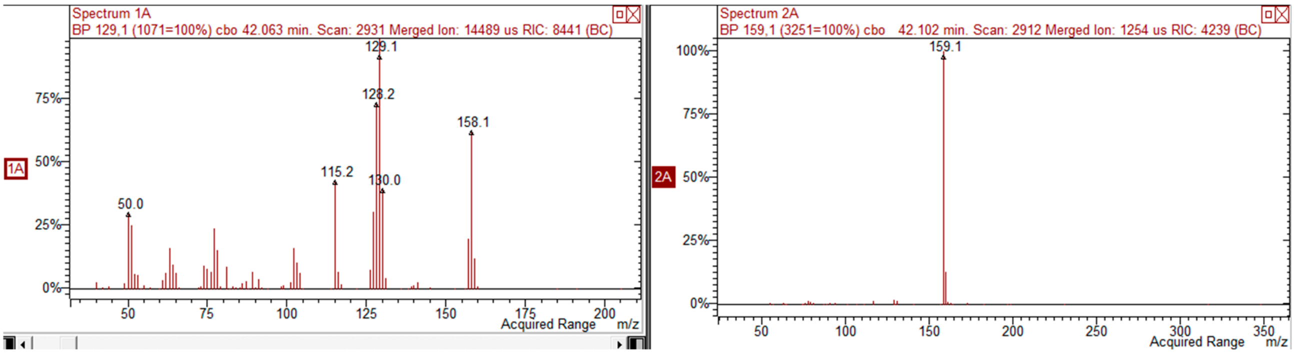

Figure S5. EI (left) and CI (right) MS spectra of unknown, time 72.28 min compounds

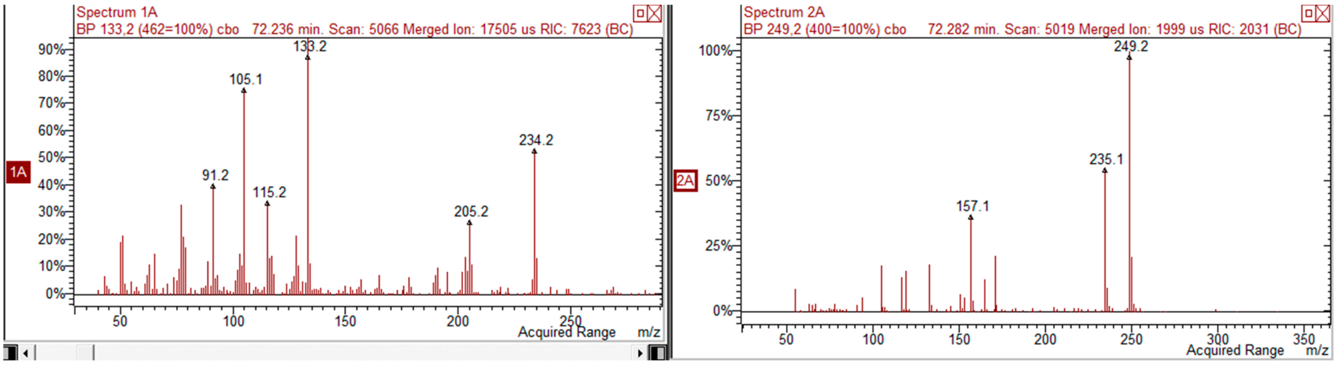

a) Time 72.28 min

Link for original GC-MS files (SMS):

https://drive.google.com/file/d/1ZOX5G7EwkiWrfbP96nj9tfsgnVKOgRf9/view?usp=sharing
